# Use of Random T-DNA Mutagenesis in Identification of Gene *UvPRO1*, A Regulator of Conidiation, Stress Response, and Virulence in *Ustilaginoidea virens*

**DOI:** 10.3389/fmicb.2016.02086

**Published:** 2016-12-27

**Authors:** Bo Lv, Lu Zheng, Hao Liu, Jintian Tang, Tom Hsiang, Jinbin Huang

**Affiliations:** ^1^Key Laboratory of Plant Pathology of Hubei Province, College of Plant Science and Technology, Huazhong Agricultural UniversityWuhan, China; ^2^School of Environmental Sciences, University of Guelph, GuelphON, Canada

**Keywords:** *Ustilaginoidea virens*, false smut, *UvPRO1*, conidiation, stress response, pathogenicity

## Abstract

False smut of rice, caused by *Ustilaginoidea virens* (Cooke) Takahashi (teleomorph: *Villosiclava virens*), is one of the most important diseases affecting rice worldwide. *Agrobacterium tumefaciens*-mediated transformation was used to identify functional genes in *U. virens*. In this study, we selected a single-copy insertion mutant T133 with deficiency in producing conidia by screening the T-DNA insertion mutant library of *U. virens*. The *UvPRO1*-deletion mutant was successfully obtained after cloning the targeted gene by analysis of the T-DNA insert site of mutant T133. Further research showed that the *UvPRO1* mutant was reduced in growth rate and could not produce conidia in PSB medium, while sensitivities to sodium dodecyl sulfate, Congo red, and hyperosmotic stress increased. Moreover, the *UvPRO1* deletion mutant hyphae could extend along the surface of spikelets at 1–3 dpi, but mycelia became shriveled and completely lost the ability to infect spikelets at 4 dpi. The relative expression level of *UvPRO1* at 8 dpi was more than twice as high as that at 1–2 dpi. These results suggest that *UvPRO1* plays a critical role in hyphal growth and conidiation, as well as in stress response and pathogenesis. These findings provide a novel mode of action for the *PRO1* protein in fungi and improve the understanding of the function of *UvPRO1* in the life cycle of *U. virens*.

## Introduction

False smut of rice, caused by *Ustilaginoidea virens* (Cooke) Takahashi (teleomorph: *Villosiclava virens*), is a minor disease that has been present in the major rice-growing areas of Asia, Africa, and America for some time (Deng, 1989; Savary et al., 2000; Ashizawa et al., 2010). Since the beginning of this century, it has become one of the most devastating grain diseases that threatens rice production worldwide, due to the widespread cultivation of susceptible high-yield hybrid rice varieties, intensive application of chemical fertilizers, and an apparent change in global climates (Rush et al., 2000; Wang et al., 2004; Singh and Pophaly, 2010; Guo et al., 2012). Occurrence of rice false smut not only affects yield, but creates a health issue by producing ustiloxins, which are microtubule inhibitors toxic to humans and animals (Koiso et al., 1994; Miyazaki et al., 2009).

Prior research on *Ustilaginoidea virens* has concentrated on the biology of the organism, including its distribution and detection, toxin production, and disease cycle and management (Zhou et al., 2003; Brooks et al., 2009; Tang et al., 2013). Compared with other important diseases such as rice blast and bacterial leaf blight, studies on the interaction of the false smut pathogen and the rice host at the molecular level are few. Sun et al. (2013) reported the genome sequence of *U. virens* and predicted possible effectors. Zhang et al. (2008) characterized the first *MAPK* protein from *U. virens* and verified that *UVMK1* is a homolog of *Magnaporthe grisea PMK1*. Rao et al. (2014) cloned a homolog of *HOG1* from *U. virens* and measured transcript levels of *UvHog1* under salinity conditions, suggesting that *UvHog1* may be involved in the specific response to salt stress. Fan et al. (2015) used time-course microscopic and transcriptional approaches to investigate host responses to *U. virens* infection, and the results implied that *U. virens* may hijack rice nutrient reservoir systems to successfully colonize rice floral organs and to form false smut balls.

In recent years, generation of random mutant collections via *Agrobacterium tumefaciens*-mediated transformation (ATMT) has been widely used in different fungal species to study gene functions (Mullins and Kang, 2001; Mullins et al., 2001; Sugui et al., 2005; Frandsen, 2011). Zhang et al. (2006) first reported the transformation of *U. virens* by the ATMT method. Yu et al. (2013) cloned the *spo76* gene in the T-DNA insertion mutant A2588, which is a high-yield mutant of rice germ, and found that reduced levels of *spo76* gene expression may enhance conidiation of *U. virens*. Yu et al. (2015) obtained 37 mutants with reproducible pathogenic defects and cloned the *UvSUN2* gene from mutant B20; their morphophysiological characterization analysis suggested that *UvSUN2* was required for hyphal growth, cell wall construction, stress response, and virulence. Wang et al. (2015) selected an avirulent T-DNA insertion mutant, B1464, and obtained a C_2_H_2_-type zinc finger protein gene, which might be related to sporulation and pathogenicity. Bo et al. (2016) found a *GH18* family gene in *U. virens* by screening of a T-DNA insertional library, which is most likely related to hyphal growth, sporulation, and pathogenicity. Zheng M.T. et al. (2016) cloned and analyzed *Uvt3277*, which is a low-affinity iron transport protein, verifying the relationship with pathogenicity by RNAi.

Although previous research studies have reported many genes which might be related to hyphal growth, sporulation, or pathogenicity, few studies of deletion targeted genes by homologous recombination have been reported in *U. virens*. It may be possible that *U. virens* has a relatively low homologous recombination frequency, as so far only Zheng D. et al. (2016) obtained the *UvHOG1* deletion mutant and demonstrated that *UvHOG1* likely has a conserved role in regulation stress responses, hyphal growth, and possibly secondary metabolism.

In this study, we selected four strains of sporulation defect mutants and one strain that does not produce a conidia by screening the T-DNA insertion mutant library, and we successfully obtained a *UvPRO1* deletion mutant after cloning the target gene by analysis of the T-DNA insert site of mutant T133. Further research showed the *UvPRO1* mutant was reduced in for growth rate and conidiation, and had increased sensitivity to sodium dodecyl sulfate (SDS), Congo red (CR) and hyperosmotic stress, and significantly reduced virulence. However, the *PRO1* gene has not been reported in *U. virens*; it was first identified in *Sordaria macrospora* in a genetic screen for mutations defective in perithecia development (Masloff et al., 1999, 2002).

In *Cryphonectria parasitica*, disruption of the *PRO1* gene resulted in a significant reduction in asexual sporulation and loss of female fertility (Sun et al., 2009). Tanaka et al. (2013) identified a mutant with an insertion in *PRO1* in a forward genetic screen to identify *Epichloe festucae* symbiosis genes, and demonstrated that *PRO1* is a central regulator for *in planta* specific growth of *E. festucae*. Compared with the role of *PRO1* in other fungi, *UvPRO1* not only regulated hyphal growth and conidiation, but was also involved in stress response and pathogenesis. Functional elucidation can provide a novel mode of action of *PRO1* in fungi and improve our understanding of the function of *UvPRO1* in the life cycle of *U. virens*.

## Materials and Methods

### Strains, Plasmids, and Plants

The wild-type strain HWD2 and all the transformants of *U. virens* generated in this study were routinely cultured on potato sucrose agar (PSA, 2% sucrose plus extract from boiled peeled potato) at 28°C, and stored in the form of mycelial-colonized filter paper at -20°C. The *A. tumefaciens* strain EHA105 and binary vector pTFCM were used for *U. virens* transformation. Plasmids KS1004 and pneoP3300III were used for gene disruption or complementation vector construction.

The susceptible rice cultivar Wanxian 98 was used in virulence assays. The seeds were kept for 24 h at 30°C before planting. After 10 days, four seedlings were placed into pots (25 cm × 20 cm × 30 cm, length × width × height) each containing 5 kg of autoclaved paddy soil. In the greenhouse, pots were fertilized twice (4 g carbamide per bucket): once at tillering (after 45 days of growth) and just before inoculation at the at the booting stage (after 90 days of growth; Jia et al., 2015).

### *Agrobacterium*-Mediated Transformation of *U. virens*

*Agrobacterium*-mediated transformation was carried out following the protocols described Yu et al. (2015) with minor modifications. The wild-type strain HWD2 was cultured in a 250 mL flask containing 150 mL liquid potato sucrose broth (PSB). The flask was placed in a shaking incubator at 28°C in the dark. After shaking at 160 rpm for 7 days, the cultures were filtered through multiple layers of cheese cloth, and conidia were obtained from the filtrate by centrifugation (3,000 rpm for 5 min). The conidial suspension was adjusted to 1 × 10^6^ conidia per mL using a haemocytometer.

The *A. tumefaciens* strain EH105 was grown at 28°C with shaking at 180 rpm for 48 h in minimal medium supplemented with kanamycin (50 μg/mL). Then, *A. tumefaciens* cells were grown in induction medium supplemented with 200 μM acetosyringone. After shaking at 180 rpm for an additional 10 h at 28°C, bacterial cultures were diluted to an optical density of 0.5 OD units at 600 nm and were mixed 1:1 with a conidial suspension from HWD2 (10^6^ spores/mL). The mix was plated onto co-cultivation medium with a layer of nitrocellulose filter. After co-cultivation at 24°C for 4 days, the membrane was removed, and placed mycelium-side down onto PSA containing 500 μg/mL of cefotaxime to counter-select bacteria, and 200 μg/mL of hygromycin to select for *U. virens* transformants. After incubation at 28°C for 5–7 days, transformant colonies were transferred to PSA plates containing 200 μg/mL of hygromycin for a second round of selection.

To test for the mitotic stability of the integrated hygromycin resistance cassette, 20 randomly chosen transformants were cultivated on PSA without hygromycin. After weekly transfer to new plates for four passages by subculturing of hyphal tips, transformants were grown on PSA plates containing hygromycin (200 mg/mL).

### Conidiation Test of ATMT Transformants

The fungus was propagated in PSA plates for 14 days at 28°C. Then, 3-mm-diameter mycelia dishes were cut from the edge of a colony and inoculated in a 50 mL flask containing 30 mL PSB which was placed in a shaking incubator. After shaking at 180 rpm for 7 days, the cultures were filtered through three layers of gauze, and conidial production was measured using a haemocytometer. The experiment was repeated three times with three replicates each time.

### Amplification and Analysis of T-DNA Flanking Sequences

Genomic DNA sequences of the transformants flanking T-DNA insertions were amplified by TAIL-PCR (thermal asymmetric interlaced-polymerase chain reaction) and inverse PCR with primer sequences shown in Supplementary Table 1. For TAIL-PCR, genomic DNA was used as a template in successive reactions with nested left border primers (LB1, 2, and 3) and right border primers (RB1, 2, and 3) together with the degenerate primers (AD1, 2, 3, or 4). PCR settings for TAIL-PCR followed Liu et al. (2013). For inverse PCR, genomic DNA was digested with SacI and circularized with T4 DNA ligase (Invitrogen, Karlsruhe, Germany). The product was purified using a Nucleic Acid Purification kit (Axygen, Union City, CA, USA). The reaction conditions for first round PCR were: 1 cycle at 95°C for 5 min, 30 cycles of 95°C for 30 s, 55°C for 45 s, and 72°C for 4 min and a final cycle at 72°C for 5 min. The second round nested PCR was performed with the same PCR program using 1 ml of the first round PCR product (diluted 1: 50) as a template together with nested primers (Liu et al., 2013). Flanking sequences recovered by TAIL-PCR and inverse PCR were analyzed with the BLAST tool hosted by the National Center for Biotechnology Information^1^ against the GenBank database and the genome sequences of *U. virens* (NCBI, JHTR00000000.1). Nucleotide sequences were compared with known protein sequences using BLASTX (NCBI^2^). Open reading frames (ORFs) were analyzed using FGENESH (Softberry Inc., Mount Kisco, NY, USA), conserved domains were detected by comparison to the Conserved Domain Database of NCBI^3^.

### Identification and Disruption of the *U. virens* PRO1 Gene

The full sequence of *UvPRO1* was obtained from the genome sequence of *U. virens* (NCBI, JHTR00000000.1). To confirm sequence presence, the primers *UvPRO1*F and *UvPRO1*R (Supplementary Table 1) were designed and used for the amplification of the *UvPRO1* gene from HWD2 isolates. Primers were all designed using Primer Premier 5.0^4^, and ORFs were analyzed using FGENESH. Protein domain and motif predictions were performed with SMART software^5^.

The *PRO1* protein sequences from different organisms were obtained from the GenBank database, using the BLAST algorithm with the *UvPRO1* sequence. Sequence alignments were performed using the Clustal X (version 2.0^6^), and a phylogenetic tree was generated with Mega software (version 7.0^7^) using the Neighbor-Joining method.

To assess the function of *UvPRO1*, which was potentially mutated in T133, a vector was constructed for the targeted disruption of *UvPRO1* by means of homologous recombination. Vector KS1004 was constructed by cloning a 1.9 kb PtrpC-hph cassette into the SmaI site of pBluescriptII KS, and the hygromycin resistance was used as the first selectable marker for screening of disruption transformants. Vector pneoP3300III was generated by cloning a 2.1 kb neomycin resistant gene cassette into the XbaI site of pCAMBIA3300, and the neomycin resistance was used as the second selectable marker.

A pair of gene-specific primers, *UvPRO1*F1F and *UvPRO1*F1R (Supplementary Table 1), was used to amplify the 900 bp fragment (**Figure 4A**) in the 5′ coding region of *UvPRO1*. Another pair of gene-specific primers, *UvPRO1*F2F and *UvPRO1*F2R (Supplementary Table 1), was used to amplify the 978 bp fragment, containing part of the 3′ coding region of *UvPRO1* (**Figure 4A**). The 900 bp HindIII/SalI-fragment (5′ region of *UvPRO1*) and the 978 bp XbaI/KpnI-fragment (3′ region of *UvPRO1*) were cloned into the corresponding restriction sites of the vector KS1004, resulting in the preliminary vector KS1004-*UvPRO1*. The hph-*UvPRO1* cassette (with a 900 bp HindIII/SalI-fragment, a 1909 bp hph-fragment, and a 978 bp XbaI/KpnI-fragment) was cloned into pneoP3300III, resulting in the gene disruption vector p3300neo*UvPRO1*.

This vector, p3300neo*UvPRO1*, was transformed into *A. tumefaciens* EHA105 by electroporation, and then hyphae were transformed with the ATMT protocol. To find *UvPRO1* disruption transformants, cultures were grown on PSA amended with hygromycin (200 mg/mL), and then subcultured onto PSA amended with 800 μg/mL of antibiotic G418 (Amresco, Solon, OH, USA). Gene disruption transformants were subjected to PCR with two pairs of primers, *UvPRO1*KF/*UvPRO1*KR and HphF/HphR (Supplementary Table 1), and amplicons were detected by PCR and Southern blot analysis.

### Complementation of *UvPRO1* Disruption Mutant

To confirm targeted gene disruption, the disruption mutant Δ*UvPRO1*-27 was complemented with a full length sequence of *UvPRO1*. Because *UvPRO1* disruption mutants were unable to grow on the PSA supplemented with G418, the neomycin resistance cassette was chosen as a selectable marker for the complementation transformation. The complementation plasmid p3300neo*UvPRO1*-Com was based on pneoP3300III. The 3,315 bp *UvPRO1* fragment (*UvPRO1* ORF plus 574 bp 5′-flanking and 905 bp 3′-flanking sequences) was amplified from genomic DNA of the wild-type with the primer pair *UvPRO1*ComF and *UvPRO1*ComR (Supplementary Table 1), and cloned into the BamHI site of pneoP3300III to generate the complementation plasmid p3300neo*UvPRO1*-Com. To obtain the *UvPRO1* complementation transformants, Δ*UvPRO1*-27 was transformed with vector p3300neo*UvPRO1*-Com by the ATMT method. The complementation transformants were screened on PSA containing 800 μg/mL G418, and gene fragments were detected by RT-PCR.

### DNA Manipulation and Southern Blot Analysis

Genomic DNA was extracted using CTAB (Sambrook et al., 1989). For Southern blot analysis of T-DNA insertion in *U. virens*, PCR was used to confirm the presence of T-DNA insertions by using primers HphF and HphR (Supplementary Table 1) to amplify an 887 bp internal region of the hygromycin resistance gene (hph). DNA from the wild-type and the transformants was completely digested with SacI, which has only one recognition site in the binary vector pTFCM, and then size-fractionated through a 0.8% agarose gel and mounted onto a positively charged nylon membrane (**Figure 2A**). The hph gene was excised from the pTFCM vector and labeled with digoxigenin (DIG)-dUTP using the PCR DIG probe synthesis kit (Roche, Mannheim, Germany) following manufacturer’s instructions. Hybridization was detected using a DIG luminescence detection kit. For Southern blot analysis of *UvPRO1* disruption mutants, genomic DNA from the wild-type and the putative *UvPRO1* disruption mutants were digested with SacI at 37°C for 24 h. The nylon membrane was hybridized with probe P (**Figure 4A**).

### RNA Isolation and qRT-PCR Analysis

Hyphae harvested from PSB medium were collected at different points in time (3, 4, 5, 6, 7, 8, and 9 days), as well as inoculated spikelets at different points in time (1, 2, 3, 4, 6, 8, 10, and 12 days). These were frozen in liquid nitrogen and stored at 80°C until required. RNA was extracted using a TRIzol Plus RNA purification kit (Invitrogen, Carlsbad, CA, USA). DNA contamination was removed by DNaseI treatment (RNase free; TaKaRa, Dalian, China). First-strand cDNA was synthesized by using a RevertAid^TM^ first strand cDNA synthesis kit (Fermentas, St. Leon-Rot, Germany). Expression of *UvPRO1* in disruption mutants and the complementation strain were examined by RT-PCR, and a 1560-bp fragment was amplified with gene-specific primers qRT-*UvPRO1*F and qRT-*UvPRO1*R (Supplementary Table 1). PCR conditions used 25 cycles of 94°C for 30 s, 58°C for 30 s, and 72°C for 1 min, with a final extension at 72°C for 5 min.

Expression of *UvPRO1* at different developmental stages of the fungus *in vitro* or *in planta* was analyzed by qRT-PCR with *UvPRO1* gene-specific primers qRT-PRO1F/qRT-PRO1R (Supplementary Table 1). PCR conditions were 40 cycles of 94°C for 15 s, 55°C for 20 s, and 72°C for 15 s, and with a final extension from 65°C to 95°C (0.5°C/5 s; Gu et al., 2012). The *U. virens*α-tubulin2, as the reference gene, was amplified with primers α-tubulin2F and α-tubulin2R (Supplementary Table 1). PCR reactions were run on a PTC-200 DNA Engine Peltier thermal cycler (BioRad, Hercules, CA, USA). The whole experiment was repeated three times.

### Phenotypic Analysis

For mycelial growth, mycelial plugs (5 mm in diameter) were transferred from 12-day-old PSA plates and grown on fresh PSA medium at 28°C. After 6 and 12 days of being cultured, the radial growth of vegetative mycelia was measured. For conidial production, strains were grown in PSB medium at 28°C. After shaking at 180 rpm for different lengths of time (4, 5, 6, 7, 8, and 9 days), the cultures were filtered through three layers of gauze, and conidial production was measured using the haemocytometer. For testing the sensitivity to various stress chemicals, the strains exposed to CM medium containing either exogenous 0.1–0.5 M NaCl, 0.01–0.05% SDS, or 30–70 mg/L CR were assessed also by measuring colony diameter of 14-day cultures. Each treatment was repeated three times.

### Pathogenicity Assay

For pathogenicity analysis, mycelial plugs of the wild-type, *UvPRO1* knock out and complementation strains were transferred from 12-day-old PSA plates and grown in PSB medium at 28°C. After shaking at 180 rpm for 5 days, the cultures were homogenized in a blender, and rice plants were inoculated with 2 mL of mycelial suspension using a syringe in the middle section of distal internodes at the eight stage of panicle development. The rice plants were placed in a plant growth chamber (Wuhan Ruihua Instrument and Equipment Co., Ltd., Wuhan, China) equipped with a high pressure sodium lamp (12 h light/dark cycle) with conditions set at a RH of 95 ± 5% and a temperature of 25 ± 1°C. After a post-inoculation surface wetness period of 120 h, plants were transferred to a greenhouse equipped with an automatic climate control system set at 28 ± 2°C and 75 ± 7% RH. This experiment was repeated three times (Jia et al., 2015). Five of the injected panicles were sampled at each time point (1, 2, 3, 4, 6, 8, 10, and 12 days after inoculation) and others were used to count the severity of false smut infection 15 days after inoculation.

### Scanning Electron Microscopy

The samples for scanning electron microscopy were first fixed with 2.5% (v/v) glutaraldehyde in 50 mM phosphate buffer (pH 7.2) for 6–8 h at 4°C, before a rinse with the same buffer for 2 h. They were then fixed in 1% (w/v) osmium tetroxide in 50 mM phosphate buffer for 1 h. After dehydration in a graded acetone series, the samples were critical-point dried, mounted on stubs, sputter coated with gold-palladium, and viewed using a JEOL JSM-6390LV scanning electron microscope operating at 10 kV (Hu et al., 2014).

### Statistical Analysis

The quantitative data were analyzed with DPS statistical analysis software (version 3.01, China Agric. Press, Beijing, China), using ANOVA. When significant treatment effects were found (*P* < 0.05), separation of means was done using Fisher’s Least Significant Difference test.

## Results

### Screening and Analysis of Sporulation Deficiency Mutants

Using the modified protocol for ATMT, a total of 3,016 hygromycin-resistant transformants of *U. virens* were obtained. The mitotic stability of the integrated T-DNA was tested by analysis of 20 randomly selected transformants. Transformants were serially subcultured for five time on PSA medium not containing hygromycin. Transformants retained the integrated T-DNA, as indicated the ability to grow on PSA containing hygromycin.

**FIGURE 1:**
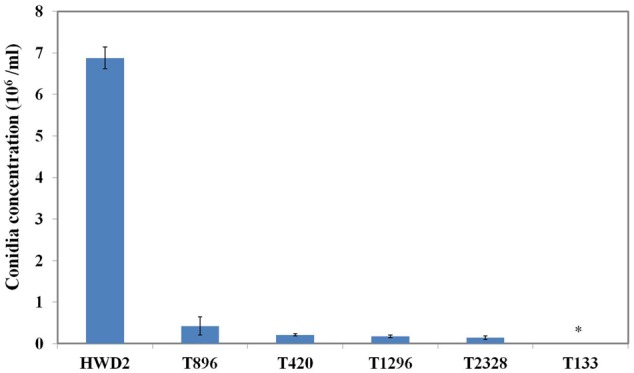
**Sporulation by conidiation defect mutants.** Quantitative analysis of the conidiation of *Ustilaginoidea virens* wild-type HWD2 and five mutants following growth for 7 days at 28°C in potato sucrose broth (PSB) medium. Asterisks indicate no conidia were observed in T133.

**FIGURE 2:**
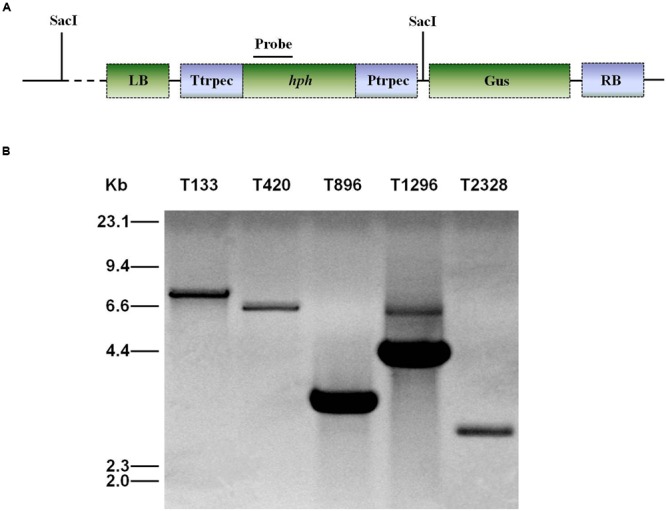
**Southern blot analysis of genomic DNA of *U. virens* mutants. (A)** The top section shows the ATMT vector pTFCM. The region for the probe used for Southern blot hybridization and SacI restriction sites are indicated. **(B)** The bottom section shows Southern blot analysis of conidiation mutants of *U. virens*. Genomic DNA of transformants was digested with SacI.

All of the 3,016 transformants were screened for sporulation deficiency and five transformants with sporulation deficiency were found. Four transformants (T420, T896, T1296, T2328) were found to have significantly (*P* < 0.05) lower conidial production, and one transformant (T133) was found to have no conidia (**Figure 1**). Southern blot analysis showed that, among several mutants with sporulation deficiency, four (T133, T420, T896, T2328) contained a single T-DNA insertion and T1296 contained two T-DNA copies (**Figure 2B**). T-DNA flanking sequences were recovered from these mutants by amplifying genomic DNA sequences flanking T-DNA insertions of transformants with TAIL-PCR and inverse PCR (Supplementary Table 1). These sequences were used to screen the GenBank database and the genome sequences of *U. virens* (NCBI, JHTR00000000.1). FGENESH was used to identify ORFs around the T-DNA insertion site. ORF sequences were compared against protein sequences from NCBI with BLASTX. Details on affected genes and disruption sites are shown in **Table 1**.

**Table 1:** Summary of *Ustilaginoidea virens* genes identified from T-DNA flanking sequences with the best BLAST matches.

Mutant	Insertions^a^	T-DNA insertion^b^	Best BLAST match with functional annotation			
			Putative function (NCBI accession)	Query coverage	*E*-value	Organism
T-133	1	In ORF	Transcriptional regulatory protein (XP_007812494.1)	100%	9e-127	*Metarhizium acridum*
T-420	1	Upstream	Hypothetical protein (KOM18477.1)	44%	1e-30	*Ophiocordyceps unilateralis*
T-896	1	In ORF	Ser/Thr protein phosphatase (XP_007807767.1)	65%	2e-102	*Metarhizium acridum*
T-1296	2	Upstream	Hypothetical protein (WP_004893973.1)	33%	9.6	*Acinetobacter schindleri*
		Unknown				
T-2328	1	Upstream	Polysaccharide synthase Cps1 (KLO79232.1)	94%	0	*Fusarium fujikuroi*

*^a^Number of insertion sites determined by Southern blot hybridization following SacI digestion of genomic DNA of transformants. A Sad site is present in the T-DNA but outside the hph probe. ^b^Locations of T-DNA insertion sites. Positions relative to the Open reading frame (ORF) show distance upstream of predicted start codon or downstream of predicted stop codon.*

In mutant T420, the targeted gene encodes a hypothetical protein, showing similarity to a protein of unknown function from *Ophiocordyceps unilateralis* (GenBank KOM18477.1). In mutant T896, a single insertion was located inside a predicted ORF of a gene with significant similarity to the Ser/Thr protein phosphatase gene of *Metarhizium acridum* (GenBank XP_007807767.1). In mutant T1296, one insertion was located upstream of a gene that showed high sequence similarity to a hypothetical protein from *Acinetobacter schindleri* (GenBank WP_004893973.1), and the other failed during cloning. In mutant T2328, T-DNA targeted upstream of a gene with significant similarity to a polysaccharide synthase *Cps1* gene of *Fusarium fujikuroi* (GenBank KLO79232.1). In mutant T133, T-DNA was inserted into a predicted ORF of a *PRO1* gene encoding C6 transcription factor that showed high sequence similarity to a *PRO1* gene of *M. acridum* (GenBank XP_007812494.1; **Table 1**). The mutant T133 was characterized by no sporulation and contained a single T-DNA insertion. Therefore, our subsequent work focused on the gene *UvPRO1* in the mutant.

### Identification and Characterization of *UvPRO1*

**FIGURE 3:**
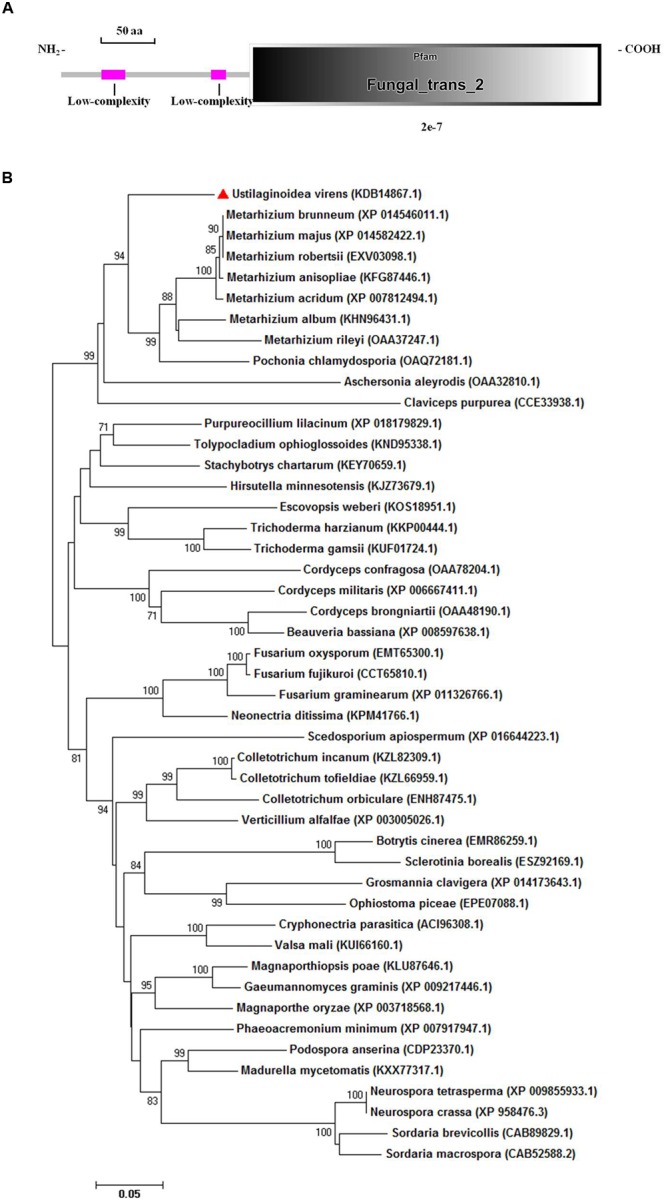
**Functional domain identification and phylogenetic tree. (A)** A conserved Fungal specific transcription factor domain (Fungal_trans_2 domain) and two low-complexity regions in *UvPRO1* were predicted using SMART website. **(B)** Neighbor-Joining analysis of *UvPRO1* with 46 homologs from other fungal species. Sequence alignments were performed using the Clustal X 2.0 program and the tree was generated using Mega 7.0 program with 1,000 bootstrap replicates. All 47 protein sequences of the *PRO1* homologs were downloaded from the NCBI database.

The aligned sequences of overlapping DNA fragments of the *PRO1* gene amplified by PCR from *U. virens* genomic DNA and from corresponding mRNA revealed a 2,440-bp ORF. The coding domain was predicted to encode a polypeptide consisting of 611 amino acids and a high level of sequence identity (84%) with transcriptional regulatory protein *PRO1* of *M. acridum* CQMa 102. Sequence analysis with SMART revealed that *UvPRO1* contained a Fungal_trans_2 conserved domain (**Figure 3A**).

Phylogenetic analysis of *UvPRO1* (GenBank KDB14867.1) to other *PRO1* proteins (**Figure 3B**) revealed that *UvPRO1* was most similar to PRO1 proteins of *Pochonia chlamydosporia* and species of *Metarhizium* (with identities above 81%), and more distant from those of other fungi (with identities above 58%). This result indicates that *PRO1* proteins are conserved among fungi tested.

### Disruption and Complementation of *UvPRO1*

**FIGURE 4:**
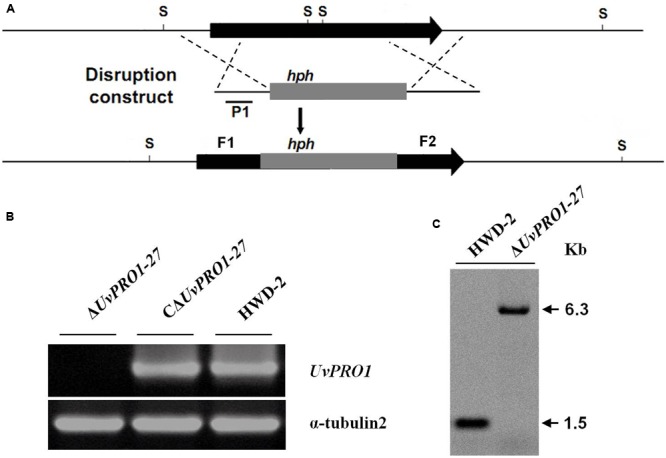
**Targeted disruption of *UvPRO1* gene. (A)** Strategic map of gene disruption vector construction and restriction map of *UvPRO1* genomic region. **(B)** RT-PCR analysis of the transcription of *UvPRO1* disruption mutant, complementation mutant, and wild-type strain with gene-specific primers *UvPRO1*F and *UvPRO1*R. **(C)** Southern blotting of the SacI-digested genomic DNA from the wild-type and *UvPRO1* disruption mutant, which was hybridized with probe P1.

A gene disruption vector, p3300neo*UvPRO1*, containing the hph gene and both the 3′ and 5′ flanking regions of *UvPRO1*, was constructed with two vectors, KS1004 and pneoP3300III (**Figure 4A**). Vector p3300neo*UvPRO1* was transformed into the wild-type, and transformants were selected on hygromycin-containing medium and on G418-containing medium. Among 628 hygromycin-resistant transformants, three without resistance to G418 were obtained.

Only one candidate disruption transformant Δ*UvPRO1*-27 was found lacking the 1208-bp *UvPRO1* fragment compared to the wild-type strain after PCR amplification with PRO1F/PRO1R (Supplementary Table 1); however, an 887-bp hph fragment was obtained by PCR amplification with hphF/hphR (Supplementary Table 1) in this candidate transformant. Furthermore, Southern blot analysis showed that single integration events had occurred in selected *UvPRO1* knockout transformant Δ*UvPRO1*-27 (**Figure 4C**), which had the 6.3-kb SacI fragment, while the wild-type strain HWD2 had the 1.5-kb SacI fragment. Null mutation of the *UvPRO1* gene was further confirmed by RT-PCR analysis, since the *UvPRO1* transcript was not detected in the targeted disruption transformant. These results demonstrated that the *UvPRO1* gene was deleted in the *UvPRO1* disruption transformant Δ*UvPRO1*-27. To investigate whether altered growth phenotypes and the loss of virulence in *UvPRO1* disruption transformants could be restored by reintroduction of a wild-type copy of *UvPRO1*, we transformed Δ*UvPRO1*-27 with plasmid pNeo3300III*UvPRO1*-Com. Subsequently, complementation transformant CΔ*UvPRO1*-27 was confirmed by RT-PCR analysis (**Figure 4B**) and was selected for further studies.

### *UvPRO1* Affects Vegetative Growth and Conidiation

**FIGURE 5:**
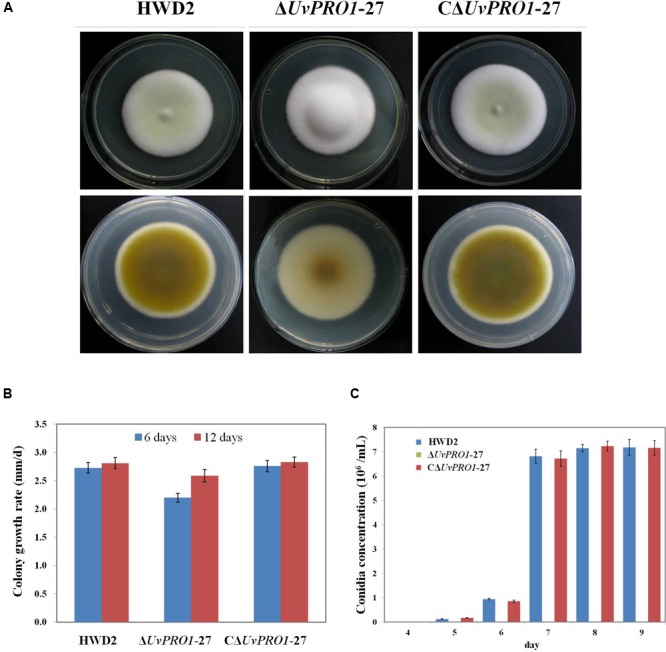
**Growth phenotypes of wild-type HWD2, Δ*UvPRO1*-27, and CΔ*UvPRO1*-27. (A)** The morphology of the strains on PSA medium after incubation at 28°C for 14 days. **(B)** The growth rate of wild-type HWD2, Δ*UvPRO1*-27, and CΔ*UvPRO1*-27 on potato sucrose agar (PSA) medium. **(C)** Sporulation of wild-type HWD2, Δ*UvPRO1*-27, and CΔ*UvPRO1*-27 at different times in potato sucrose broth (PSB) medium. Asterisks indicate no conidia were observed in Δ*UvPRO1*-27, in contrast to wild-type HWD2 and CΔ*UvPRO1*-27.

The morphology of the strains was monitored on PSA medium. The Δ*UvPRO1*-27 mutant produced white colonies with long and abundant aerial hyphae, in contrast with the colonies with a light yellow center surrounded by a white edge of the wild-type rescued strain CΔ*UvPRO1*-27 (**Figure 5A**). Furthermore, the Δ*UvPRO1*-27 strain had a reduced apical extension rate (2.6 mm/d), producing smaller colonies than the wild-type (2.8 mm/d) and CΔ*Uv-PRO1*-27 (2.8 mm/d), and the mycelial growth rate measured at 6 days on PSA of the Δ*UvPRO1*-27 strain (2.20 mm/d) was significantly less than the wild-type (2.73 mm/d; **Figure 5B**).

In PSB medium, mycelia from wild-type strains and the rescued strain CΔ*UvPRO1*-27 produced hyphae with conidiophores at their tips after 5–6 days, and conidia were produced after 7 days at 6.7 or 6.8 × 10^6^ conidia/mL, respectively. However, mycelia of the Δ*Uv-PRO1-27* produced hyphae without conidiophore formation at 6 days, and no conidia were observed up to 9 days (**Figure 5C**).

### The Importance of *UvPRO1* for Regulation Responses to Hyperosmotic and Cell Membrane Stresses

**FIGURE 6:**
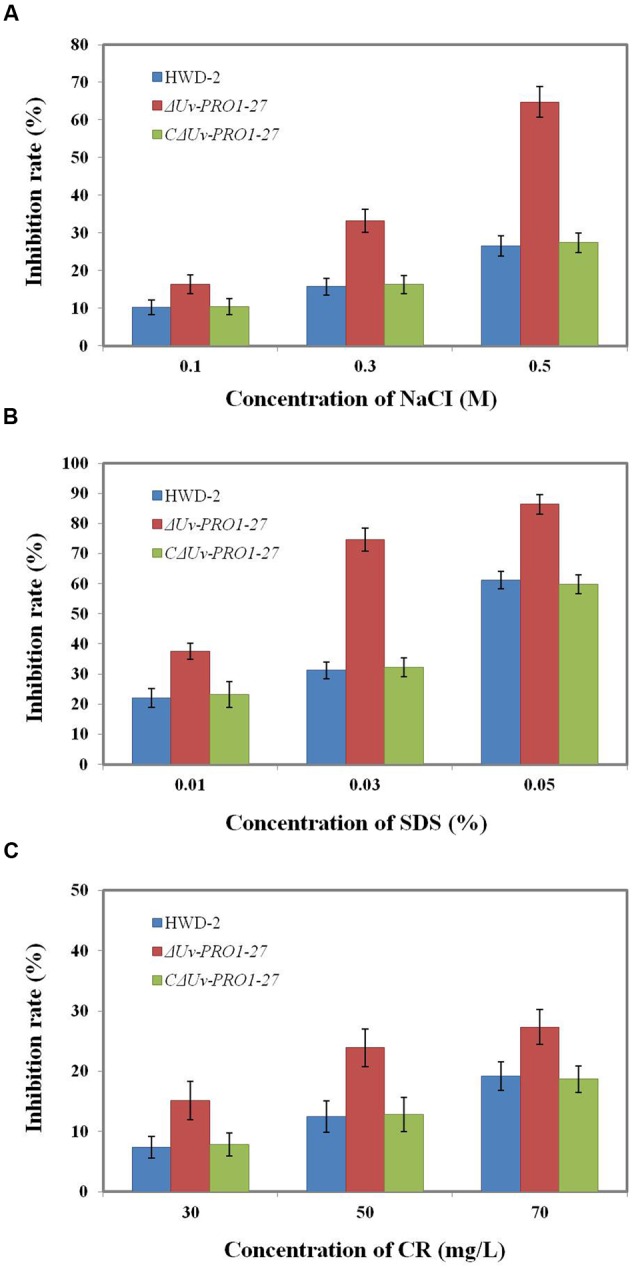
**Growth of the *UvPRO1* mutant in the presence of different stresses. (A)** The wild-type strain HWD2, Δ*UvPRO1*-27, and CΔ*UvPRO1*-27 were cultured on CM medium with 0.1–0.5 M NaCl after incubation at 28°C for 14 days. **(B)** The wild-type strain HWD2, Δ*UvPRO1*-27, and CΔ*UvPRO1*-27 were cultured on CM medium amended with 0.01–0.03% sodium dodecyl sulfate (SDS) and incubated at 28°C for 14 days. **(C)** The wild-type strain HWD2, Δ*UvPRO1*-27, and CΔ*UvPRO1*-27 were cultured on CM medium with 30–70 mg/L CR and incubated at 28°C for 14 days.

Because the mycelial growth of the *UvPRO1* mutant was interrupted, we further monitored the effects of hyperosmotic and cell membrane stresses on CM medium with 0.1–0.5 M NaCl, 0.01–0.05% SDS, or 30–70 ug/mL CR. In the presence of 0.1–0.5 M NaCl, the growth rate of all strains decreased, and Δ*UvPRO1*-27 mutant displayed more sensitivity under salt stress compared to the wild-type and CΔ*UvPRO1*-27, and the growth rate of Δ*UvPRO1*-27 mutant was reduced by 16–65%, respectively (**Figure 6A**). These results suggested that the *UvPRO1* may play a role in regulation response to hyperosmotic conditions in *U. virens*.

We also assayed the effects of SDS and CR treatments that mimic cytoplasm membrane and cell wall stresses, respectively. On CM with 0.01, 0.03, or 0.05% SDS, the growth rate of the Δ*UvPRO1*-27 mutant was, respectively, reduced by 37.5, 74.6, and 86.4%, while the decrease was 22.1, 31.5, and 61.3% in the wild-type and 23.2, 32.2, and 59.8% in CΔ*UvPRO1*-27 (**Figure 6B**). In the presence of 30–70 mg/L CR, similar results to growth assays with SDS were obtained, in that Δ*UvPRO1*-27 mutant displayed a slower radial growth rate than the wild-type or CΔ*UvPRO1*-27 (**Figure 6C**). These results suggested that the *UvPRO1* mutant also had increased sensitivity to CR and SDS. Therefore, *UvPRO1* may be involved in regulating responses to membrane and cell wall stresses in *U. virens*.

### The Effect of *UvPRO1* on the Pathogenicity of *U. virens*

**FIGURE 7:**
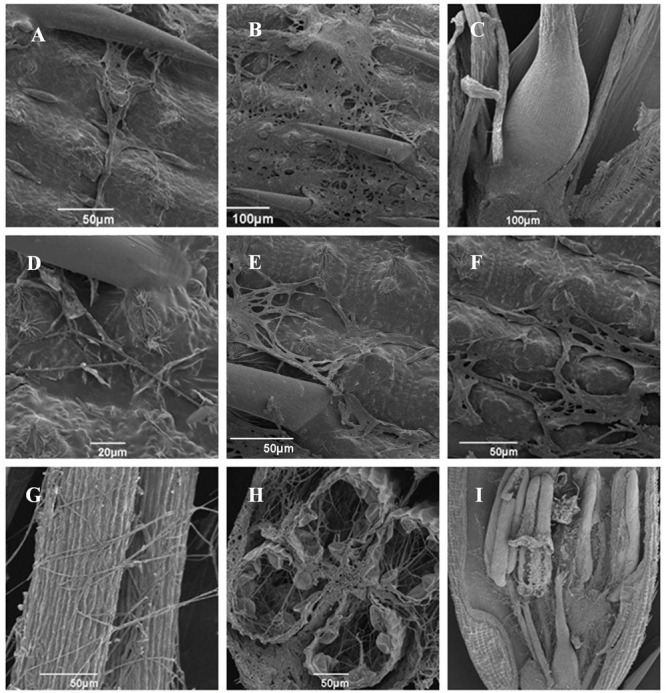
**Infection and colonization of rice spikelets by HWD2 and Δ*UvPRO1*-27at different time points. (A)** Hyphae of Δ*UvPRO1*-27 growing and extending along spikelet surface at 1–3 days post-inoculation (dpi). **(B)** Mycelia of Δ*UvPRO1*-27 dehydrated and failed to thrive on spikelets at 4–6 dpi. **(C)** Floral organs were not infected by Δ*UvPRO1*-27 until 12 dpi. **(D–F)** Hyphae of HWD2 were observed to be elongated and extending along the surface of spikelets at 1–3 dpi. **(G,H)** Mycelia of wild-type strain HWD2 were observed on the inner surfaces of spikelets, and filaments were infected by masses of mycelia at 5 dpi. **(I)** The florets were covered with many hyphae with white mycelia of HWD2 wrapped around stamens and pistils at 7 dpi.

**FIGURE 8:**
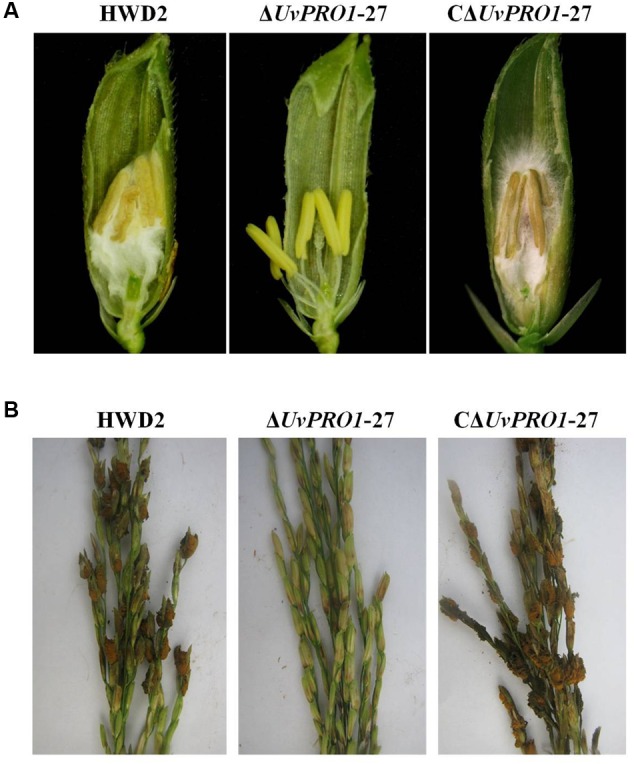
**Comparison of the pathogenicity of HWD2, Δ*UvPRO1*-27, and CΔ*UvPRO1*-27 on rice spikelets. (A)** Floral organs were not infected by *UvPRO1* at 9 dpi, but the spaces in the spikelets were filled by white mycelia of HWD2 and the *UvPRO1* complementary strain at 9 dpi. **(B)** The pathogenicity phenotype of *U. virens* on rice spikelets at 15 dpi.

Pathogenicity assays of the wild-type strain, Δ*UvPRO1* mutant, and *UvPRO1* complementary strain were performed on a susceptible host (Wanxian 98). Since the Δ*UvPRO1* mutant produced no conidia, we also used a mycelial suspension of the wild-type strain and a *UvPRO1* complementary strain for inoculation by injection as well as Δ*UvPRO1* mutant. The inoculated plants were examined for colonization and infection by *U. virens* until 12 dpi. At 1–3 dpi, for the wild-type strain HWD2 and *UvPRO1* complementary strain, many hyphal strands were observed to be elongated and extended along the surface of the spikelets (**Figures 7D–F**). At 4–6 dpi, hyphae were observed on the inner surfaces of spikelets, and filaments were infected by masses of hyphae (**Figure 7G**). At 7–8 dpi, the florets were covered profusely by hyphal growth with some wrapped around stamens and pistils (**Figures 7H,I**). At 9–12 dpi, the spaces in the spikelets were filled up by white mycelia (**Figure 8A**), and large mycelial masses grew out of the spikelets forming smut balls. After 15 dpi, 88.6% of the wild-type strain inoculated plants developed typical symptoms of false smut (**Figure 8B**), and similarly 86.2% in the *UvPRO1* complementary strain (**Table 2**). For the Δ*UvPRO1* mutant, at 1–3 dpi, hyphae were observed to be elongated and extended along the surface of spikelets (**Figure 7A**), which was similar to that of the wild-type strain and *UvPRO1* complementary strain. At 4–6 dpi, mycelia became dehydrated and failed to grow further on spikelets (**Figure 7B**). No hyphae were observed inside of spikelets until 15 dpi (**Figures 7C** and **8A,B**). This indicated that the Δ*UvPRO1* mutant lost the ability for invasive growth on spikelets, and that the *UvPRO1* is important for the pathogenicity of *U. virens*. Therefore, we conclude that the *UvPRO1* plays an important role in virulence of *U. virens*.

**Table 2:** Smut ball production by *U. virens* wild-type HWD2, and *UvPRO1* deletion and complementation mutants.

Strain	Average disease (%)	Number of average smut balls per spikelets
HWD2	88.6	30.4
Δ*UvPRO1*-27	0	0
CΔ*UvPRO1*-27	86.2	32.2

### Expression Dynamics of the *UvPRO1* Gene

**FIGURE 9:**
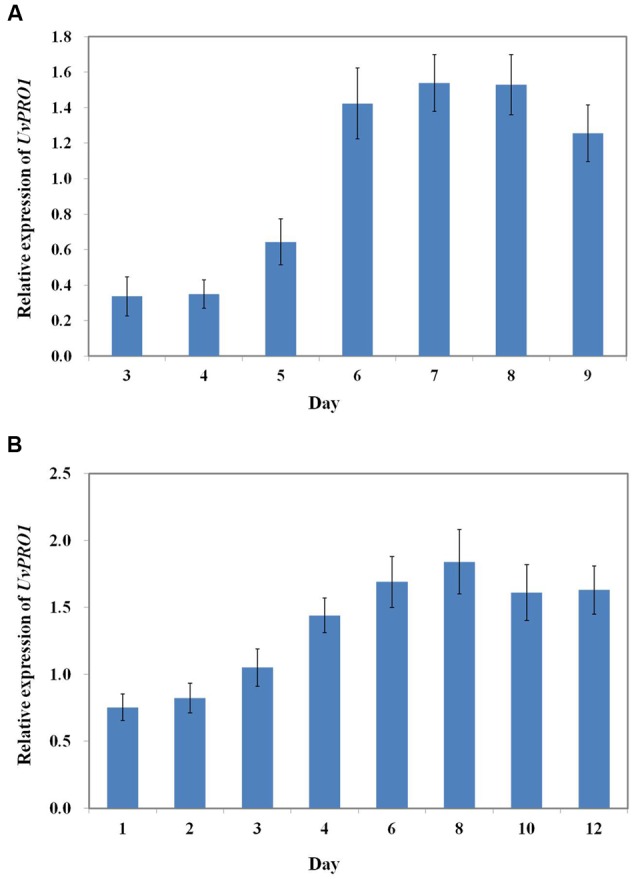
**Expression dynamics of the *UvPRO1* gene. (A)** Expression of *UvPRO1* during conidiation. An agar plug (3 mm in diam.) of wild-type strain placed into PSB medium. Values are relative to tubulin gene expression in RNA isolated from mycelia 3–9 dpi. **(B)** Expression of *UvPRO1* during infection of rice spikelets. Rice spikelets were inoculated with mycelia of either the wild-type or the *UvPRO1* deletion mutant. Values are relative to tubulin gene expression in RNA isolated from spikelets 1–12 dpi. Boxes and bars represent averages and standard error, respectively, of three independent biological replicates.

We first evaluated the *UvPRO1* expression levels of *U. virens* in PSB medium using qRT-PCR. The results showed that lower expression levels were detected during early vegetative growth stages between 1 and 5 days, and were significantly increased during the conidiation stage between 6 and 9 days (**Figure 9A**). *UvPRO1* expression during spikelet infection stage (4–12 dpi) was much higher than that in the early developmental stages (1–3 dpi), while the relative expression levels of *UvPRO1* at 8 dpi was more than twofold higher than that at 1–2 dpi (**Figure 9B**).

## Discussion

The ATMT system has been used as an effective tool for insertional mutagenesis and homologous replacement in many phylogenetically diverse fungi (Mullins and Kang, 2001; Khang et al., 2005; Frandsen, 2011; Paz et al., 2011). Many target genes have been identified by screening phenotype and pathogenicity defective mutants from fungal T-DNA random insertion mutant library with homologous replacement and complementary methods (Munch et al., 2011; Giesbert et al., 2012; Xu and Chen, 2013; López-Pérez et al., 2015). In this study, we obtained five sporulation defective mutants by screening 3,016 strains of *U. virens* T-DNA insertion mutants. Southern blot analysis revealed that 80% of *U. virens* transformants contained single-copy T-DNA insertions, which is greater than the frequency described in a previous study (Yu et al., 2015). Therefore, the ATMT system used in this study was stable and reliable, and it could provide appropriate experimental material for screening targeted genes (Maruthachalam et al., 2011; Cai et al., 2013).

Among the conidiation defective mutants, T133 showed a decrease in mycelial growth and complete loss of conidiation. Sequence analysis showed that the mutant T133 has a T-DNA insertion in a predicted ORF encoding the amino acid sequence with high similarity (84%) to *PRO1* of *M. acridum* which included the typical GAL4-like Zn(II)_2_Cys_6_ binuclear cluster DNA-binding domain.

Transcription factors of the Zn(II)_2_Cys_6_ binuclear cluster DNA-binding domain class, to which *PRO1* belongs, are the most abundant class of transcription factors in fungal genomes (Borkovich et al., 2004). Most of the characterized members of this family participate in regulation of the primary and secondary metabolic pathways, but several have been shown to regulate fungal developmental processes (Vienken et al., 2005). *PRO1* was first identified in *S. macrosporea* in a genetic screen for mutations defective in perithecial development, and gene deletion and complementation studies showed that *SmPRO1* is required for sexual development. In *C. parasitica*, deletion of *PRO1* resulted in a significant reduction in asexual sporulation and loss of female fertility (Sun et al., 2009). In *E. festucae*, Tanaka et al. (2013) identified a mutant with an insertion in *PRO1*, and disruption of targeted gene increased asexual sporulation and reduced cell fusion.

In this study, morphological observation of the *UvPRO1* deletion mutant showed that *UvPRO1* deficiency led to a decline in the hyphal growth rate, an increase in aerial hyphae, and a complete loss of sporulation. In contrast, the deletion *PRO1* gene of *Alternaria brassicae* led to a similar effect on mycelial growth in that the mycelial growth rate of the *AbPRO1* deletion mutant declined by 25% (Cho et al., 2009). Moreover, in *C. parasitica, PRO1* gene deletion also resulted in production of few or no conidia and increased aerial hyphae, but the radial growth rate was not influenced (Sun et al., 2009). Therefore, by comparing the previous research findings, the *PRO1* gene can be seen to participate in regulation of pathogen growth and development, but the role of *PRO1* in different fungi was visibly different. In addition, the *PRO1* gene has not been reported to be regulated in response to various environmental stresses such as oxidative and cell wall stresses. However, in our study, the *PRO1* deletion mutant showed increased sensitivities to hyperosmotic and cell wall stresses, which provide a novel regulation of *PRO1* in among pathogenic fungi.

In previous studies, *PRO1* has been verified to be important for pathogen virulence in *A. brassicicola* and stable maintenance of hypovirus infection *C. parasitica*. To assess the role of *UvPRO1* in virulence, we observed the infection process of the *UvPRO1* mutant and wild-type after inoculation at rice booting stage. The results showed that hyphae of the *PRO1* deletion mutant could extend along the surface of spikelets at 1–3 dpi, but mycelia became dehydrated and completely lost the ability to infect spikelets after 4 dpi. The qRT-PCR analysis of *UvPRO1* showed that the expression levels of *UvPRO1* in the infection stage (4–12 dpi) were much higher than that in early developmental stages (1–3 dpi), while the relative expression levels of *UvPRO1* at 8 dpi was more than twice as high as that at 1–2 dpi. Therefore, we conclude that the *UvPRO1* plays an important role in virulence of *U. virens*.

## Conclusion

The *UvPRO1* gene in *U. virens* was characterized as a Zn(II)_2_Cys_6_ transcription factor required for fungal developmental processes. The results of this study suggest that *UvPRO1* plays a critical role in hyphal growth and conidiation, and is also involved in stress responses and pathogenesis, which provided novel actions of *PRO1* in fungi and has improved the understanding of the function of *UvPRO1* during the life cycle of *U. virens*.

## Author Contributions

Conceived and designed the experiments: BL and JH. Performed the experiments: BL. Analyzed the experiment data: BL, LZ, HL, and JT. Contributed reagents/materials/analysis tools: BL and TH. Wrote the paper: BL. All authors have read and approve the final manuscript.

## Conflict of Interest Statement

The authors declare that the research was conducted in the absence of any commercial or financial relationships that could be construed as a potential conflict of interest.

## Footnotes

1 http://www.ncbi.nlm.nih.gov/BLAST/

2 http://www.ncbi.nlm.nih.gov

3 http://www.ncbi.nlm.nih.gov/Structure/cdd/cdd.shtml

4 http://www.premierbiosoft.com/primerdesign/

5 http://smart.embl-heidelberg.de/

6 http://www.clustal.org/clustal2/

7 http://www.megasoftware.net/index.php
