# Supplementary material for: Use of Random T-DNA Mutagenesis in Identification of Gene UvPRO1, A Regulator of Conidiation, Stress Response, and Virulence in Ustilaginoidea virens
Source: Front Microbiol. 2016 Dec 27;7:2086. doi: 10.3389/fmicb.2016.02086 (PMC5186764; doi:10.3389/fmicb.2016.02086)
Supplement: Supplementary file 1 [file Table_1.DOC]

**Supplemental Table 1**

**Primers sequences used for polymerase chain reaction in this study.**

| **Primer** | **Sequence (5` to 3`)** |
| --- | --- |
| *UvPRO1*F | TCTGGCTCCACGAACACG |
| *UvPRO1*R | AGCACTACGACCGCCTACC |
| *UvPRO1*F1F | AAGCTT GCAACTGCTCAAAGCGAGTGGTA |
| *UvPRO1*F1R | GTCGAC GGAGGTGTCTGATAGGTGCTGAA |
| *UvPRO1*F2F | GCTCTAGA AGGATTCCCTACAATGCCAACGG |
| *UvPRO1*F2R | GGGGTACC GAAGCGATACAGGAGGTAAAAGC |
| *UvPRO1*KF | CTGTCGCTGGCTGAGGAA |
| *UvPRO1*KR | GGAGTCGAGGCACGCAAT |
| HphF | TTCTGCGGGCGATTTGTG |
| HphR | AGCGTCTCCGACCTGATG |
| *UvPRO1*ComF | TTAGGATCC CTCTGGCTCCACGAACACG |
| *UvPRO1*ComR | AAAGGATCC AGCACTACGACCGCCTACC |
| ProbeF | TTGAACAAGACACCGCACAG |
| ProbeR | CGAGACAGAGTTTTGGAGGG |
| qRT-*UvPRO1*F | CTCAGTCAGCCCGCCTCCTA |
| qRT-*UvPRO1*R | TCTCGCACCACAGTCACCA |
| α-tubulin2F | AGGTTGCGTTGAAGGAGGTT |
| α-tubulin2R | GAGGTGGAGTTGCCGATAAA |
